# Fluorescence imaging using indocyanine green to identify sentinel lymph nodes during surgery for breast cancer (INFINITE): protocol for a hybrid effectiveness-implementation trial using a stepped-wedge cluster design

**DOI:** 10.1136/bmjopen-2026-117847

**Published:** 2026-06-24

**Authors:** Isabelle J Henskens, Tessa L Dinger, Iza Stekelenburg, Lea M Dijksman, Helena M Verkooijen, Emily L Postma, Annemiek Doeksen, Henk J Schuijt, INFINITE Study Group, A Doeksen

**Affiliations:** 1 Department of Oncological Surgery, St Antonius Hospital, Nieuwegein, Utrecht, The Netherlands; 2 Department of Research and Innovation, St Antonius Hospital, Nieuwegein, Utrecht, The Netherlands; 3 Imaging Division Trial office, University Medical Centre Utrecht, Utrecht, The Netherlands; 4 Utrecht University, Utrecht, The Netherlands; 5 Department of Internal and Geriatric Medicine, Amsterdam University Medical Centres, Amsterdam, Noord-Holland, The Netherlands; 6 Department of Surgery, St Antonius Hospital, Utrecht, The Netherlands

**Keywords:** Implementation Science, Breast surgery, Breast tumours, Breast imaging

## Abstract

**Introduction:**

Sentinel lymph node biopsy (SLNB) is the standard procedure for axillary staging in clinically node-negative breast cancer. Traditionally, SLNB is performed using technetium-labelled (^99m^Tc) nanocolloid, with or without blue dye. However, both tracers have important limitations. Blue dye poses safety risks, while ^99m^Tc-nanocolloid introduces additional hospital visits, radiation exposure, logistical complexity and high costs. Indocyanine green (ICG) fluorescence is a non-radioactive alternative, offering real-time visualisation while addressing many limitations of traditional tracers. Yet, adoption of ICG-guided SLNB remains limited. This trial aims to guide the implementation of ICG-guided SLNB via axillary incision, evaluate its real-world effectiveness and inform conditions for nationwide scale-up.

**Methods and analysis:**

The INFINITE trial is a multicentre, hybrid effectiveness-implementation study employing a stepped-wedge cluster design across seven Dutch hospitals. Clusters sequentially transition from SLNB using ^99m^Tc-nanocolloid alone (Phase I) to ICG as the primary tracer and ^99m^Tc-nanocolloid as a within-patient control (Phase II), and finally to ICG alone (Phase III). The hybrid design enables evaluation of implementation outcomes (penetration, adoption, fidelity, appropriateness, feasibility, acceptability), intervention outcomes (effectiveness, safety, costs) and patient-reported experience (patient satisfaction). The primary outcome is penetration, the proportion of Phase III SLNB procedures performed with ICG alone. An integrated implementation approach combines the Grol and Wensing model (process model), the Consolidated Framework for Implementation Research (determinant framework) and Proctor’s outcomes framework (evaluation framework). Outcomes are assessed quantitatively, supplemented by an embedded mixed-methods component to explain variation in implementation across centres.

**Ethics and dissemination:**

Ethical approval was obtained from the Medical Research Ethics Committees United (6 November 2024; NL87551.100.24). Results will be submitted to open-access, peer-reviewed journals and presented at conferences focused on oncological or image-guided surgery. Implementation tools, including a clinical protocol, implementation guide, educational materials and patient information, will be developed to support national adoption.

**Trial registration number:**

NCT07146295.

**STRENGTHS AND LIMITATIONS OF THIS STUDY**

- The hybrid effectiveness-implementation design enables simultaneous evaluation of implementation outcomes (penetration, adoption, fidelity, appropriateness, feasibility and acceptability), intervention outcomes (effectiveness, safety and costs) and patient-reported experience (patient satisfaction).
- The stepped-wedge cluster design supports iterative evaluation and progressive optimisation of implementation strategies across diverse hospital settings.
- Implementation tools, including a clinical protocol, an implementation guide, educational materials and patient information, will be developed, tested and refined during the trial to support national scale-up of indocyanine green-guided sentinel lymph node biopsy.
- Cluster transitions are based on site readiness rather than random allocation, which may reduce control over potential time-related confounding when comparing the pre-implementation (Phase I) and post-implementation (Phase III) cohorts.
- Questionnaire-based implementation outcomes (appropriateness, feasibility and acceptability) are assessed at a single time point in Phase III, which may limit characterisation of how these outcomes evolve during implementation.

## Introduction

 Sentinel lymph node biopsy (SLNB) is the standard axillary staging procedure for patients with clinically node-negative breast cancer, guiding both prognosis and adjuvant treatment decisions.^1^,^4^ The current gold standard approach is a radio-guided surgery using technetium-labelled (^99m^Tc) nanocolloid. This technique requires preoperative injection and lymphoscintigraphy to localise the sentinel lymph node (SLN), followed by intraoperative detection with a gamma probe.^5^ To enable visualisation, ^99m^Tc-nanocolloid may be combined with blue dye.^6 7^ This dual-tracer approach is endorsed in the Dutch National Guidelines due to its association with lower false-negative rates.^8^

Despite their diagnostic performance, both traditional tracers have important limitations. ^99m^Tc-nanocolloid exposes patients and staff to ionising radiation, requires coordination with nuclear medicine services and necessitates at least one additional preoperative hospital visit, often at a different facility due to limited availability. Its short half-life restricts surgical planning, and, as a radioisotope, it incurs high costs. The radioactive signal can interfere with tumour markers, and production raises environmental and ethical concerns related to uranium mining.^9^,^13^ Blue dye carries a risk of severe allergic reactions and permanent skin discolouration.^14 15^ The limited added diagnostic value of combining ^99m^Tc-nanocolloid with blue dye often does not justify the increased burden, leading to the common practice of using ^99m^Tc-nanocolloid alone.^16^,^18^ Collectively, these drawbacks highlight the need for alternative techniques.

Fluorescence imaging using indocyanine green (ICG) has emerged as a safe and effective alternative that addresses many limitations of traditional tracers.^19^ ICG is injected intraoperatively after induction of general anaesthesia and enables real-time visualisation of lymphatic drainage using near-infrared fluorescence (NIRF) imaging.^20 21^ The INFLUENCE trial, a previous non-inferiority study, demonstrated that ICG-guided SLNB via axillary incision provided higher SLN detection rates than ^99m^Tc-nanocolloid, with equivalent pathological SLN detection.^22^ Its efficacy has been supported by multiple studies and systematic reviews.^2023^,^32^ A recent meta-analysis of 39 studies (4236 procedures) demonstrated that ICG is superior to blue dye and non-inferior or potentially superior to ^99m^Tc-nanocolloid alone or in combination with blue dye. These results were consistent across per-case and per-node detection rates, sensitivity for metastatic nodes and the number of nodes retrieved.^33^

Beyond diagnostic performance, ICG offers additional advantages to patients, healthcare providers and society. It eliminates preoperative hospital visits and the need for injections while the patient is awake.^23^ As a fluorescent dye, ICG does not depend on specialised nuclear medicine facilities, simplifying logistics and surgical planning. Unlike ^99m^Tc-nanocolloid, ICG does not interfere with tumour markers and provides real-time visual feedback, which may reduce procedure time. Compared with blue dye, ICG has a favourable safety profile and is not associated with permanent skin staining.^26 34 35^ Additionally, ICG-guided SLNB has been associated with lower procedural costs and may contribute to more sustainable and resource-efficient healthcare practice.^36 37^

Despite the growing body of evidence and clear advantages, nationwide uptake of ICG-guided SLNB remains limited and most hospitals continue to rely on ^99m^Tc-nanocolloid. For many years, SLNB has been embedded in a radio-guided workflow using a handheld gamma probe. Transitioning to ICG-guided SLNB replaces this long-standing technique with a camera-based system. This requires hospitals to obtain NIRF imaging equipment, adapt established operative workflows and train surgical teams. Although favourable results with ICG have been reported in the literature, surgical techniques depend on operator skill and confidence and are therefore less readily adopted than pharmacological interventions.^38^,^40^ The transition to ICG may be accompanied by a technical learning curve, underscoring the need to manage early adoption in a manner that preserves oncological safety. Structured implementation support is essential to ensure patients benefit equitably and to mitigate unwarranted practice variation. The INFINITE trial therefore aims to implement ICG-guided SLNB via axillary incision, evaluate its real-world effectiveness and inform the conditions for nationwide scale-up.

## Methods and analysis

### Aims

The primary aim of the INFINITE trial is to implement ICG-guided SLNB via axillary incision into routine clinical practice. Secondary aims are to identify key barriers and facilitators to implementation and to generate evidence to inform conditions for nationwide adoption and future consideration for inclusion in the Dutch national breast cancer guidelines.

### Study design and setting

#### Study design

The INFINITE trial is a multicentre, hybrid effectiveness-implementation study employing a stepped-wedge cluster design.^41^ The hybrid design enables concurrent evaluation of implementation outcomes, intervention outcomes and patient-reported experience, while the stepped-wedge design facilitates phased adoption of ICG-guided SLNB across participating hospitals. Quantitative analyses are complemented by an embedded mixed-methods component to capture contextual and process-related factors relevant to implementation.

**Figure 1:**
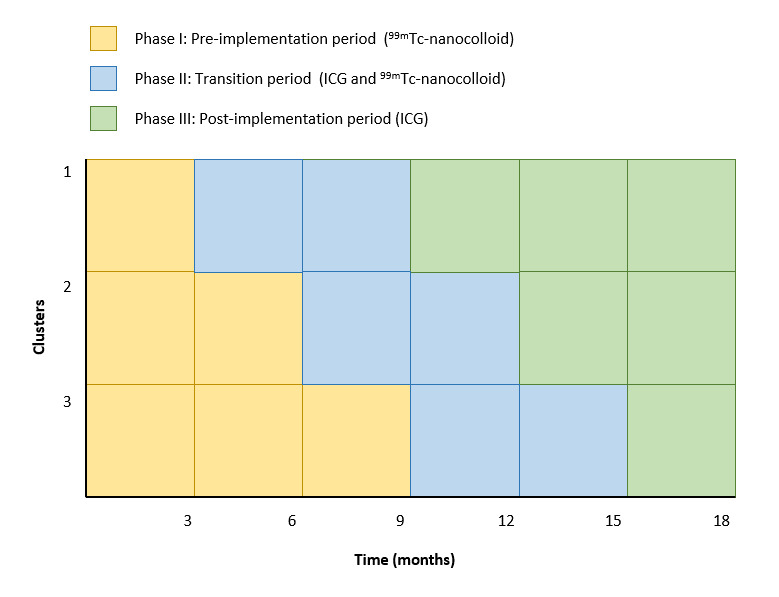
INFINITE stepped-wedge cluster trial. Clusters: cluster 1 (Diakonessenhuis, Ziekenhuisgroep Twente), cluster 2 (Dijklander Ziekenhuis, Noordwest Ziekenhuisgroep, Alrijne) and cluster 3 (Canisius Wilhelmina Ziekenhuis, Spaarne Gasthuis). ICG, indocyanine green; ^99m^Tc, radioisotope technetium-99m.

Clusters implement ICG-guided SLNB across three sequential phases (figure 1). Phase I (pre-implementation period) is an observational cohort study reflecting current practice in which SLNB is performed using ^99m^Tc-nanocolloid as per standard of care. Phase II (transition period) is a fixed 6-month period in which SLNB is guided primarily by ICG, with concurrent use of ^99m^Tc-nanocolloid as a within-patient control to support a safe transition. Phase III (post-implementation period) is an observational cohort study representing the new practice, with SLNB performed using ICG as a single tracer.

The stepped-wedge cluster design allows for iterative evaluation at each transition. Process evaluations conducted at these intervals will inform adaptation and optimisation of the implementation approach (see an integrated implementation approach). This strategy aligns with the Idea, Development, Exploration, Assessment, Long-term study (IDEAL) framework for surgical innovation, which recognises that surgical innovations often require context-specific refinement during adoption.^38^,^40^

#### Participating hospitals

**Figure 2:**
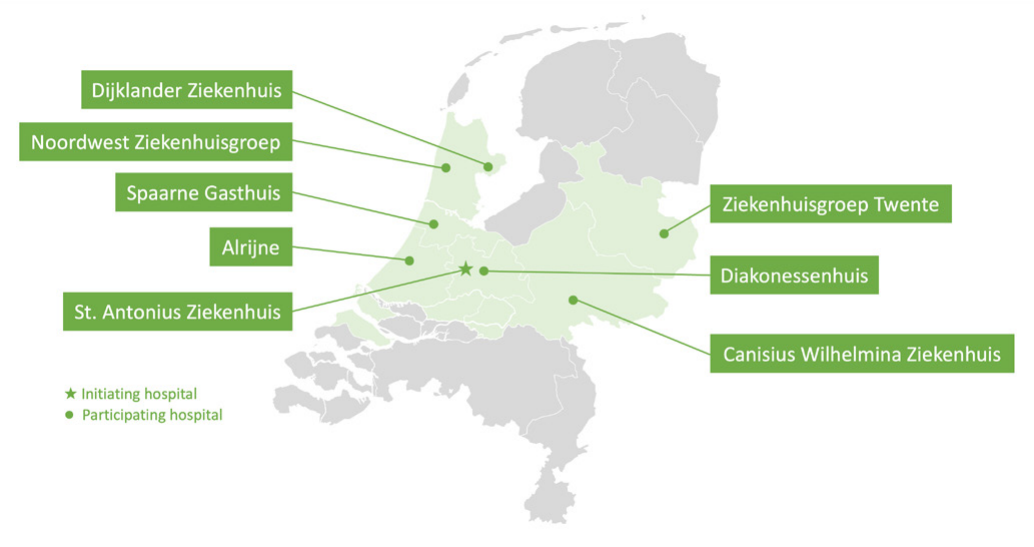
Participating and initiating hospitals.

Seven Dutch hospitals were purposively selected to represent a range of geographic regions, institutional types (eg, OncoMid, Santeon) and organisational scales. This selection captures diversity in implementation context and enhances the relevance of findings for national scale-up (figure 2).

#### Timeline and cluster transition

All participating hospitals commenced Phase I on 18 April 2025. The trial spans 18 months and is divided into six consecutive 3-month periods. Three clusters, each comprising two or three hospitals, transition sequentially from Phase I to Phase II at 3-month intervals.

Cluster transitions are not randomised but scheduled pragmatically based on site readiness, defined as the availability of ICG, access to a NIRF imaging system and completion of training. Although non-randomised transitions may reduce control over potential time-related confounding, the relatively short study duration and stable clinical context minimise the likelihood of major secular trends, making this approach appropriate for a pragmatic implementation study.

### Study population

Patients aged ≥18 years with ductal carcinoma in situ (DCIS) or biopsy-confirmed invasive T1–T3 breast cancer are eligible if clinically node-negative as confirmed by preoperative axillary ultrasound and if there is an indication for breast surgery with SLNB via axillary incision. Exclusion criteria include combined marking of the axillary node with a radioactive iodine seed procedure, known allergy to ICG, iodine or intravenous contrast agents, prior axillary lymph node dissection (ALND), hyperthyroidism or thyroid cancer, pregnancy or breast-feeding and absence of informed consent.

### Training

During Phase I, all participating sites receive a training programme, comprising an e-learning module, an in-person workshop and a train-the-trainer cascade. Training is evaluated through anonymous participant feedback, which is used to refine the e-learning module, in-person workshop and educational materials. Where feasible, all surgeons performing SLNB via axillary incision for breast cancer will participate directly. Alternatively, a train-the-trainer model may be applied, for example, in hospitals where SLNB is performed by a limited number of dedicated breast surgeons, who subsequently train colleagues locally. Before entering Phase II, each cluster must confirm site-level implementation readiness.

### Transition period

The fixed 6-month transition period is designed to accommodate the anticipated learning curve and is informed by prior research and implementation experience, including the coordinating hospital’s successful adoption of ICG as a single tracer following the INFLUENCE trial.^22^ When SLNB using ^99m^Tc-nanocolloid and blue dye was first implemented as an alternative to ALND, early reports described higher false-negative rates during the introductory phase.^42 43^ Subsequent studies showed that these learning-curve effects could be mitigated through structured training programmes, standardised protocols and on-site monitoring.^44^

In contrast, ICG-guided SLNB modifies an established surgical procedure rather than introducing a completely new one. Most procedural steps remain unchanged, and real-time fluorescence offers intuitive visualisation of the SLN, thereby limiting the need for extensive retraining. Empirical evidence on its learning curve remains limited, deriving mainly from two recent studies in which ICG was introduced alongside traditional tracers.^45 46^ Neither study evaluated transition to ICG as a single tracer, and both also included SLNB performed through the mastectomy incision, for which dedicated evidence remains limited. These studies therefore provide indirect, but reassuring, insights into the learning curve of ICG-guided SLNB via axillary incision.

A 6-month transition period was selected to provide sufficient opportunity for surgeons across participating hospitals to safely gain experience with ICG while retaining ^99m^Tc-nanocolloid as a within-patient control. This duration accommodates variation in surgical volume, prior experience with fluorescence-guided surgery and team composition, ranging from dedicated breast surgery teams to mixed surgical services. It also allows sufficient case accrual to achieve performance stabilisation before reliance on ICG alone. A fixed transition period promotes progression across sites, while three-monthly evaluations enable timely identification of sites requiring additional training, mentoring or support.

### ICG administration

ICG is reconstituted with sterile water for injection to obtain a 2.5 mg/mL (0.25% or 3.2 mM) solution. A total dose of 5 mg (2 mL) is injected intra- and subdermally into two to four periareolar sites after induction of general anaesthesia but before disinfection and sterile draping. The dose and injection technique are based on the INFLUENCE trial, which demonstrated non-inferior SLN detection compared with ^99m^Tc-nanocolloid using this approach in the Dutch clinical context. This dosing regimen falls within the recommended range and maximum daily dose specified in the Summary of Product Characteristics. Periareolar intra- and subdermal injections were selected for their reproducibility and suitability for palpable and non-palpable tumours.

### SLNB per study phase

#### Phase I (pre-implementation period)

**Figure 3:**
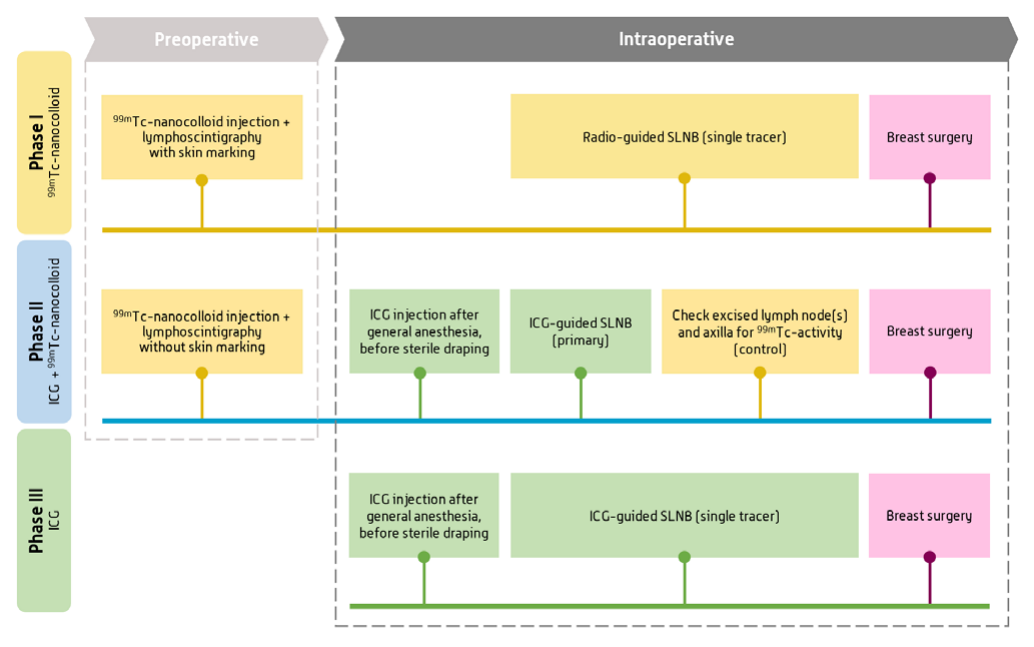
Sentinel lymph node biopsy procedure per study phase. ICG, indocyanine green; SLNB, sentinel lymph node biopsy; ^99m^Tc, radioisotope technetium-99m.

SLNB is performed using ^99m^Tc-nanocolloid, according to standard clinical practice (figure 3). At the Nuclear Medicine Department, ^99m^Tc-nanocolloid is injected the morning of surgery (70 MBq) or the preceding day (150 MBq), followed by lymphoscintigraphy and skin marking of the SLN location. Intraoperatively, the SLN is identified using a handheld gamma probe providing auditory feedback. The lymph node (LN) with the highest counts per 10 seconds is considered the SLN; if two LNs demonstrate equal counts, both are classified as SLNs.

#### Phase II (transition period)

During the transition period, both ICG and ^99m^Tc-nanocolloid are administered. Preoperative preparation at the Nuclear Medicine Department mirrors Phase I, with the exception that skin markings are omitted and surgeons are blinded to lymphoscintigraphy results. Intraoperatively, the SLNB is guided primarily by ICG, with ^99m^Tc-nanocolloid serving as a within-patient control.

ICG is administered as described in the ICG administration section. A caudal axillary incision is made 5–30 min after injection, and SLNB is performed before breast tumour resection. Lymphatic channels may be clipped to prevent ICG leakage. The SLN is defined as the LN with the highest fluorescent signal and is excised first. The axilla is subsequently inspected for residual fluorescent LNs; if present, up to two additional LNs may be excised. All excised LNs are assessed ex vivo for ^99m^Tc uptake. The axilla is then explored for residual ^99m^Tc activity using the gamma probe, supplemented by inspection and palpation. Additional LNs are excised in accordance with the predefined limit of two.

#### Phase III (post-implementation period)

SLNB is performed using ICG as a single tracer, following the same technique as described for Phase II but without concurrent use of ^99m^Tc-nanocolloid as a within-patient control. This approach eliminates the need for a preoperative visit to the Nuclear Medicine Department.

### An integrated implementation approach

The INFINITE trial employs an integrated implementation approach that combines a complementary process model, determinant framework and evaluation framework, consistent with Nilsen’s taxonomy.^47^ The implementation process is guided by the Grol and Wensing Model of Implementation of Change.^48^ Barriers and facilitators to implementation are identified using the updated Consolidated Framework for Implementation Research (CFIR).^49^ Implementation outcomes, intervention outcomes and patient-reported experience are structured using the Outcomes for Implementation Research framework by Proctor *et al.*^50^ Together, these frameworks support a theory-based, systematic and reproducible implementation strategy that may inform similar implementation efforts in other settings.

**Figure 4:**
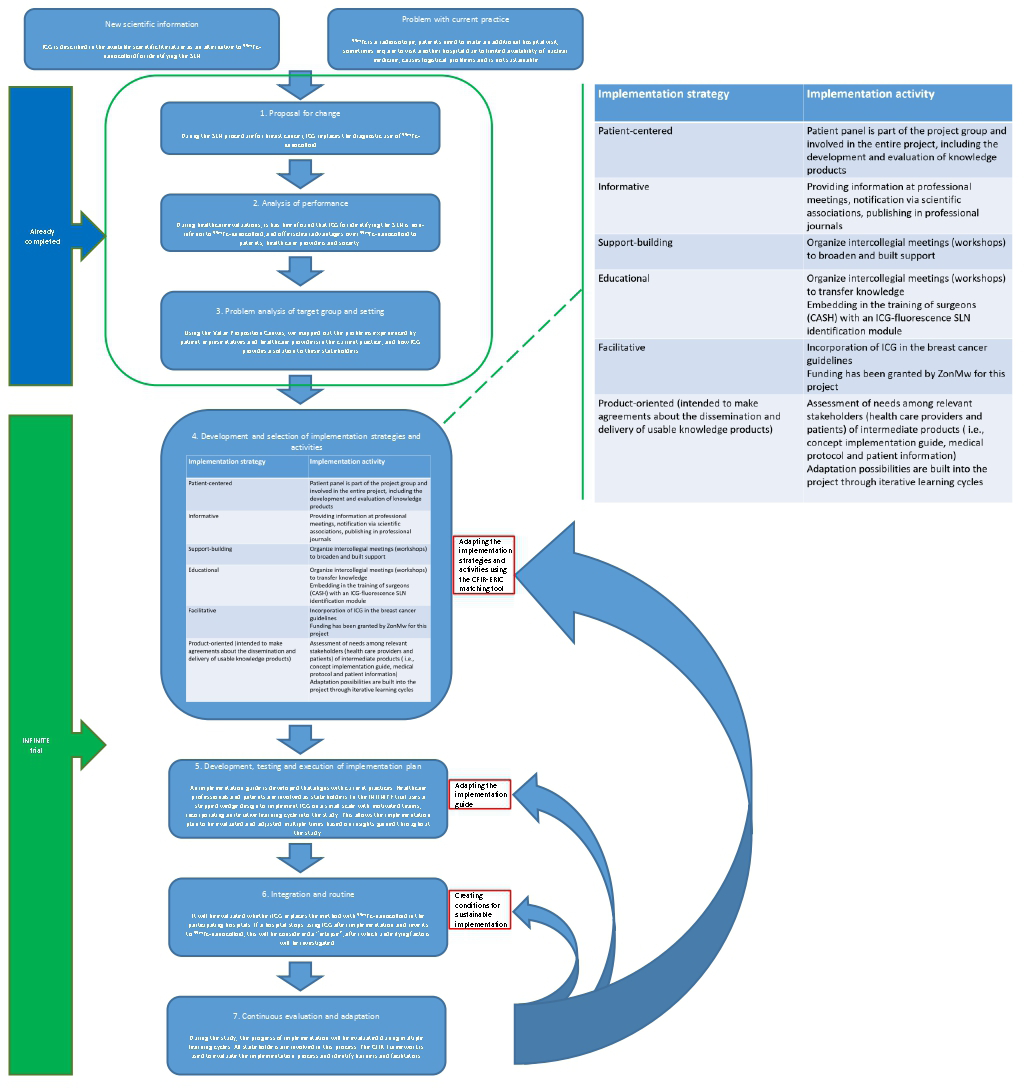
Integrated implementation approach. Combining the Grol and Wensing Model of Implementation of Change, Proctor’s outcomes framework, the Consolidated Framework for Implementation Research (CFIR) and the Expert Recommendations for Implementing Change (ERIC) with the CFIR-ERIC matching tool^48^,^51^. CASH, Cursorisch onderwijs AIOS Heelkunde (Dutch national surgical training programme for residents); CFIR, Consolidated Framework for Implementation Research; ERIC, Expert Recommendations for Implementing Change; ICG, indocyanine green; SLN, sentinel lymph node; ^99m^Tc, radioisotope technetium-99m; ZonMw, Netherlands Organisation for Health Research and Development.

The Grol and Wensing process model outlines seven, often iterative, steps to support systematic change (figure 4).^48^ Steps 1–3 were completed prior to trial initiation. In Step 1, the proposed change was to replace ^99m^Tc-nanocolloid with ICG as the tracer for SLNB via axillary incision in breast cancer. This proposal was supported by evidence from the INFLUENCE trial and a growing body of literature demonstrating non-inferiority of ICG-guided SLNB, together with several advantages compared with conventional tracers, which informed Step 2 (analysis of performance).^2022 23 26^,^33^ In Step 3, a problem analysis was conducted using the Value Proposition Canvas to systematically map the limitations of ^99m^Tc-nanocolloid and identify the unmet needs of patients and healthcare providers.

In Step 4, six initial implementation strategies were selected. Their selection was informed by the Step 3 problem analysis, complemented by determinants from the SLNB and ICG-fluorescence literature and input from the multidisciplinary project team. The strategies were operationalised using the Expert Recommendations for Implementing Change (ERIC) project and Netherlands Organisation for Health Research and Development (ZonMw) guidelines (figure 4).^51^ Patient-centred strategies involve engagement of the patient panel throughout the study, including the development and evaluation of knowledge products. Informative strategies comprise disseminating information at professional meetings, through scientific associations and in peer-reviewed journals. Support-building strategies include organising in-person workshops aimed at strengthening clinical support and stakeholder engagement, which also serve as an educational strategy by facilitating knowledge transfer and skill development. Educational strategies are further supported by embedding an ICG-guided SLNB module in the Dutch national surgical training programme for surgical residents (Cursorisch onderwijs AIOS Heelkunde, CASH). Facilitative strategies aim to support consideration of ICG-guided SLNB for inclusion in national breast cancer guidelines and are supported by ZonMw project funding. Product-oriented strategies involve identifying stakeholder needs regarding key implementation tools and developing these materials accordingly (eg, the concept implementation guide, medical protocol and patient information). These strategies may be adapted during the trial as part of ongoing iterative evaluation cycles.

Step 5 comprises the small-scale introduction of ICG during the stepped-wedge trial in motivated clinical teams, accompanied by the development and testing of a uniform medical protocol and an implementation guide. Step 6 evaluates the extent to which ICG replaces ^99m^Tc-nanocolloid for SLNB in participating sites, with penetration as the trial’s primary outcome. Step 7 entails iterative, 3-month evaluation cycles embedded in the trial. Barriers and facilitators will be identified using the updated CFIR across five domains: innovation, outer setting, inner setting, individuals and implementation process.^49^ This is predominantly a project-based activity supporting iterative optimisation, with the CFIR-ERIC matching tool used to refine implementation strategies where appropriate.^51^ The recurring evaluation cycles also allow timely identification of sites that require additional support. One CFIR-based assessment is conducted as a formal research component. This is embedded within the trial’s mixed-methods design and comprises brief open-text questions in the healthcare provider questionnaire administered in the third month of Phase III.^49^ These responses contextualise the quantitative survey outcomes.

### Outcomes and outcome measures

Following the framework of Proctor *et al,*^50^ outcomes are categorised into three domains: implementation outcomes (penetration, adoption, fidelity, appropriateness, feasibility and acceptability), intervention outcomes (effectiveness, safety and costs) and patient-reported experience (patient satisfaction). Table 1 provides an overview of all outcomes, definitions, outcome measures and data sources or instruments.

**Table 1:** Overview of implementation, intervention and patient-reported experience outcomes with corresponding definitions, outcome measures and data sources

Implementation outcomes	Definition	Outcome measure	Data source/instrument
Adoption	Intention, initial decision or action to try or employ ICG fluorescence	Proportion of hospitals performing SLNB with ICG only (Phase III) Proportion of surgeons performing SLNB with ICG only (Phase III)	Quantitative: hospital administrative data
Penetration	Integration of a practice within a service setting and its subsystems	Proportion of SLNB procedures performed with ICG only relative to all SLNBs in the target population (Phase III)	Quantitative: hospital administrative data
Fidelity	Degree to which ICG fluorescence is implemented as intended	Proportion of SLNB procedures conducted according to protocol (Phase III) Number, type and reasons for protocol deviations related to ICG preparation, storage and administration (Phase III)	Intraoperative healthcare provider questionnaire with embedded open-text fields (Phase III)
Appropriateness	Healthcare providers perceived fit, usefulness and relevance of ICG fluorescence for identifying the SLN	Perceived relevance Perceived strength of evidence Reliability of ICG fluorescence Patient-specific suitability Overall level of appropriateness	Healthcare provider questionnaire (REDCap) with embedded open-text fields (Phase III)
Feasibility	Extent to which ICG fluorescence can be successfully used within a given setting	Site readiness (availability of fluorescence imaging equipment) Ease of learning Experience with dual-tracer SLNB prior to transitioning to ICG only Confidence in transitioning to ICG only Suitability for routine clinical practice Team attitude and motivation Collaboration with nuclear medicine Overall level of feasibility	Healthcare provider questionnaire (REDCap) with embedded open-text fields (Phase III)
Acceptability	Extent to which healthcare providers perceive ICG fluorescence to be agreeable, palatable or satisfactory	Perceived relative advantage Ease of use, complexity and patient-friendliness Overall level of acceptability Preference for ICG vs ^99m^Tc-nanocolloid and underlying reason Willingness for sustained use Likelihood of recommending ICG fluorescence to colleagues	Healthcare provider questionnaire (REDCap) with embedded open-text fields (Phase III)

Intervention outcomes	Definition	Outcome measure	Data source/instrument
Effectiveness		Lymph node yield per SLNB procedure (total number of excised lymph nodes, including SLNs and non-SLNs) (all phases) Per-case SLN detection rate, defined as the proportion of SLNB procedures in which ≥1 SLN is identified by the assigned tracer (Phases I and III) Within-patient per-case SLN detection comparing ICG and ^99m^Tc-nanocolloid (Phase II) Per-node SLN detection by tracer, reported separately for ICG and ^99m^Tc-nanocolloid (all phases) Tracer concordance of SLNs (Phase II) Detection time, defined as time from axillary skin incision to excision of the first SLN (all phases) Total SLNB duration, defined as time from axillary skin incision to axillary wound closure (all phases) Pathological classification of excised SLNs (benign, isolated tumour cells, micrometastases, macrometastases), reported per node and per patient and stratified by tracer and study phase (all phases)	Quantitative: perioperative data collected by healthcare providers during SLNB and supplemented with patient file data, entered into REDCap case report forms
Safety		(Serious) adverse events potentially related to ICG administration (Phases II and III)	Quantitative: postoperative REDCap case report forms using patient file data
Costs		Cost overview analysis (healthcare provider perspective): comparison of per-patient and in-hospital costs of SLNBs using ICG vs ^99m^Tc-nanocolloid Budget impact analysis (national scale-up): projected economic impact on the Dutch healthcare system over a 5-year period through phased implementation of ICG-guided SLNB Patient-incurred costs related to SLNB, including travel, sick leave and childcare needs (Phases I and III)	Quantitative: hospital administrative data, financial data and national costing guidelines; ZonMw Budget Impact Analysis tool with sensitivity analysis; phase-specific postoperative patient questionnaire (REDCap)

Patient-reported experience	Definition	Outcome measure	Data source/instrument
Satisfaction	Satisfaction with various aspects of ICG fluorescence: content, complexity, comfort, delivery and credibility	Patients perceived satisfaction with SLNB experience using ^99m^Tc-nanocolloid, dual-tracer SLNB and ICG only	Phase-specific postoperative patient questionnaire (REDCap), with embedded open-text fields

Definitions of implementation, intervention and patient-reported experience outcomes are based on Proctor *et al.*^50^

ICG, indocyanine green; 99mTc, radioisotope technetium-99m; REDCap, research electronic data capture; SLN, sentinel lymph node; SLNB, sentinel lymph node biopsy; ZonMw, Netherlands Organisation for Health Research and Development.

These outcome domains are interrelated; the quality of implementation may influence both intervention performance and patient experience, which in turn may affect sustained uptake. Quantitative data alone are not sufficient to understand the contextual factors underlying these relationships.^52^ Therefore, the trial primarily follows a quantitative design with an embedded mixed-methods component, limited to the questionnaire-based outcomes assessing appropriateness, feasibility, acceptability and patient satisfaction. These questionnaires mainly include closed-ended items, supplemented by brief open-ended questions that allow respondents to elaborate on their responses, consistent with a concurrent one-phase mixed-methods design.^53^ The qualitative data serve a complementary and explanatory role that may contextualise the quantitative findings. The study will be reported in accordance with the Good Reporting of A Mixed Methods Study (GRAMMS) and Standards for Reporting Implementation Studies (StaRI) guidelines.^54 55^

#### Implementation outcomes

Penetration is the primary implementation outcome and is defined as the proportion of SLNB procedures performed using ICG as a single tracer during Phase III, relative to the total number of SLNB procedures performed on the target population in that phase. The numerator consists of all Phase III SLNB procedures performed with ICG only, and the denominator is derived from aggregated sources such as procedural codes.

Adoption is assessed at both hospital and provider levels. Hospital-level adoption is defined as the proportion of participating hospitals performing SLNB with ICG as a single tracer in Phase III. Provider-level adoption is the proportion of surgeons performing SLNB with ICG as a single tracer in Phase III relative to all surgeons performing SLNB. Entry into Phase II (dual-tracer use) reflects initial adoption, and exclusive ICG use in Phase III indicates full adoption. Continued dual-tracer use beyond Phase II is considered delayed adoption, and reversion to ^99m^Tc-nanocolloid is classified as relapse.

Fidelity is measured intraoperatively through surgeon self-reporting of deviations from the ICG-guided SLNB protocol and their rationale for these deviations.

Appropriateness, feasibility and acceptability are assessed using a structured healthcare-provider questionnaire administered 3 months into Phase III. Although generic instruments for implementation outcomes are available, these are not tailored to the clinical and organisational context for ICG-guided SLNB and may not adequately capture procedure-specific, workflow-related and organisational determinants relevant to this setting.^56 57^ Therefore, a context-specific questionnaire was developed using a CFIR-informed approach, supplemented by determinants derived from the Measurement Instrument for Determinants of Innovations framework and factors identified in the SLNB and ICG-fluorescence literature.^19 49 58^ Questionnaire items were designed to operationalise Proctor’s implementation outcomes in a manner relevant to the current clinical context. The instrument was co-developed by researchers and oncological surgeons with expertise in ICG fluorescence and iteratively refined based on feedback from implementation scientists and value-based healthcare experts. Pilot testing with end-users ensured clarity and validity. The final questionnaire comprises Likert-scale items, multiple-choice questions and open-ended questions, enabling concurrent collection of quantitative data with complementary qualitative insights.

#### Intervention outcomes

Intervention outcomes are evaluated quantitatively and include effectiveness, safety and costs. Effectiveness is assessed across all study phases to provide internal benchmarks during implementation and to generate real-world effectiveness data. Effectiveness outcomes include the total number of excised LNs per SLNB procedure, including number of SLNs and non-SLNs; per-case SLN detection rate, defined as the proportion of SLNB procedures in which≥ 1 SLN is identified by the assigned tracer; per-node SLN detection by tracer, reported separately for ICG and ^99m^Tc-nanocolloid; detection time from axillary skin incision to excision of the first SLN; total SLNB procedure duration and pathological outcomes of excised SLNs, reported per-node and per patient and stratified by tracer and study phase. For Phase II in which both tracers are used concurrently, tracer concordance of SLNs is additionally assessed as a measure of agreement between tracers, and a within-patient comparison of per-case SLN detection is performed.

Safety is evaluated by systematic documentation of (serious) adverse events potentially related to ICG use during SLNB.

Costs are evaluated through a per-patient direct in-hospital cost analysis comparing ICG-guided and ^99m^Tc-nanocolloid-guided SLNB, based on hospital data and national costing guidelines. In addition, a 5-year budget impact analysis (BIA) models the financial implications of phased nationwide implementation of ICG-guided SLNB from a healthcare provider perspective. Patient-level cost data, including travel, sick leave and childcare needs, will be collected via patient questionnaires during Phases I and III and may inform future societal cost evaluations.

#### Patient-reported experience

Patient satisfaction is assessed using phase-specific postoperative questionnaires evaluating patient experience with ^99m^Tc-nanocolloid, ICG or both. As existing Dutch instruments (eg, Consumer Quality Index Mammacare) are insufficiently specific to capture experiences related to tracer use for SLNB, a context-specific questionnaire was co-developed with the patient panel (see Patient and public involvement) and patient-reported experience measure (PREM) experts.^59^ The questionnaire was iteratively refined based on PREM expert and patient panel feedback and pilot-tested to ensure clarity, relevance and content validity.

### Data collection

Baseline characteristics will be recorded for both patients and healthcare providers. Patient characteristics comprise demographic and clinical variables extracted from electronic medical records. Provider characteristics include year of birth, hospital affiliation, years of surgical experience, years of experience with SLNB for breast cancer, monthly SLNB volume and prior experience with ICG-fluorescence imaging.

Implementation outcomes will be captured through intraoperative case report forms, hospital administrative records and healthcare-provider questionnaires (table 1). Intervention outcomes will be obtained intraoperatively, from electronic medical records and from hospital financial records. Patient-reported experience and patient-level cost data will be collected using phase-specific postoperative patient questionnaires.

### Sample size

A formal sample size calculation was not performed, as the INFINITE trial primarily aims to implement ICG-guided SLNB in routine clinical practice, while concurrently collecting real-world data on effectiveness. Moreover, sample size estimation for stepped-wedge cluster designs is inherently challenging due to the sequential rollout across clusters.^41^ Instead, the study uses a convenience sample of real-world data from seven purposefully selected hospitals.

The seven participating hospitals collectively perform approximately 1500 SLNBs via axillary incision annually. Over the planned study period, this corresponds to approximately 2250 SLNB procedures. Given the limited exclusion criteria and an anticipated exclusion rate of ≤10%, approximately 2025 patients are expected to be eligible. Based on practical feasibility and consent rates observed during the early study phase, approximately 850 patients are expected to be included. The estimate is informed by initial inclusion patterns and may vary modestly over time as site-specific workflow stabilises.

The anticipated sample size is considered sufficient to estimate key quantitative outcomes with acceptable precision. For the primary implementation outcome, penetration, an expected Phase III sample of approximately 280 SLNB procedures yields a 95% CI of roughly ±6 percentage points when the proportion is near 50%, with narrower intervals for lower or higher values, based on binomial approximation. For effectiveness outcomes, assuming a per-case SLN detection rate of approximately 95% in Phase III, the corresponding 95% CI would be approximately ±2–3 percentage points. This level of precision is appropriate for pragmatic hybrid effectiveness-implementation research, in which the aim is to describe real-world uptake and contextual variation rather than to formally test hypotheses.

### Statistical analysis

#### Quantitative analysis

Baseline characteristics and quantitative outcome variables will be summarised using descriptive statistics. Continuous variables will be reported as means with SD or as median with IQR, based on distribution. Distributional assumptions will be assessed by visual inspection of histograms, and homogeneity of variances will be evaluated using Levene’s test. Dichotomous and categorical data will be presented as frequencies with percentages.

Where applicable, comparisons will be performed using the Student’s t-test for normally distributed continuous variables, the Mann-Whitney U-test for non-normally distributed continuous variables, and the χ^2^ or Fisher’s exact test for categorical variables.

Penetration, the primary implementation outcome, will be reported as a proportion with corresponding 95% CIs, without formal between-group comparisons. Adoption and fidelity outcomes will be reported descriptively as proportions with accompanying counts. Safety outcomes will be reported descriptively as proportions of patients experiencing ICG-related (serious) adverse events, with corresponding 95% CIs.

Effectiveness outcomes will be analysed according to study phase. A pre–post implementation analysis will compare observational cohorts of Phase I (^99m^Tc-nanocolloid) and Phase III (ICG). For these phases, effectiveness outcomes will be reported with 95% CIs and include per-case SLN detection rates, per-node SLN detection by tracer, number of excised LNs per procedure, detection time, SLNB duration and pathological outcomes of excised SLNs. Phase II will be analysed separately using a within-patient approach. In this phase, per-node SLN detection will be reported separately for ICG and ^99m^Tc-nanocolloid, and tracer concordance will be evaluated by quantifying agreement at the LN level. In addition, a paired within-patient comparison of per-case SLN identification by ICG versus ^99m^Tc-nanocolloid will be performed. Phase II outcomes will be reported descriptively with corresponding 95% CIs, using paired statistical methods where appropriate.

A cost overview analysis will be conducted from the healthcare provider perspective, comparing per-patient in-hospital costs for SLNB using ICG versus ^99m^Tc-nanocolloid. Total per-patient costs will be calculated by multiplying unit costs by expected resource use and summing all cost components. In addition, a 5-year BIA will be performed to model the economic impact of phased nationwide implementation of ICG-guided SLNB. Sensitivity analyses will be conducted by varying key parameters, including NIRF imaging system costs and annual surgical volumes. Patient-incurred costs will be summarised descriptively by converting patient-reported data on travel, sick leave and childcare into monetary values using national unit-cost references.

All quantitative analyses will be conducted using IBM SPSS Statistics (V.26) or R (V.4.4.0). All statistical tests will be two-sided, and a p-value of <0.05 will be considered statistically significant.

#### Qualitative analysis

Open-text responses from questionnaires will be analysed using conventional content analysis, following the approach described by Hsieh and Shannon, to inductively identify and categorise recurring themes, barriers, facilitators and explanatory factors relevant to implementation and patient experience.^60^ Findings will be summarised descriptively and illustrated with representative quotations.

#### Mixed methods data integration

Quantitative and qualitative data will be collected concurrently and analysed separately. Integration will occur during the interpretation phase using joint display tables, in which qualitative findings will be used to contextualise and explain quantitative results (eg, reasons underlying tracer preferences). Joint display tables will align quantitative outcomes with illustrative qualitative data, enabling contextualisation and explanatory comparison. This approach follows established mixed-methods integration principles described by Fetters *et al.*^61^

### Patient and public involvement

Patients were actively involved throughout the design and conduct of this trial. A dedicated patient panel was established in collaboration with Stichting Buddyhuis, a Dutch peer-support organisation that matches patients with breast cancer with current or former patients.^62^ The patient panel comprises four women with lived experience of SLNB using ^99m^Tc-nanocolloid and/or ICG. As integral and equal members of the project team, the patient panel participated in a focus group using the Value Proposition Canvas to identify patients’ needs regarding SLNB and explore the perceived added value of ICG-guided SLNB.

The patient panel also co-developed and pilot-tested the patient questionnaire and reviewed patient information materials and the informed consent documents to ensure clarity, relevance and acceptability. The patient panel will continue to contribute systematically throughout the trial via iterative evaluation cycles, including the interpretation of findings relevant to patient experience.

In addition, the Patient Advisory Group of the Dutch Breast Cancer Association (Borstkankervereniging Nederland) and the Dutch Breast Cancer Research Group (Borstkanker Onderzoek Groep), consisting of patient representatives, reviewed and endorsed the current study.

### Ethics and dissemination

This study is subject to the Dutch Medical Research Involving Human Subjects Act (Wet medisch-wetenschappelijk onderzoek met mensen, WMO). Ethical review and approval were obtained from the Medical research Ethics Committees United (MEC-U; Central Committee on Research Involving Human Subjects dossier number NL87551.100.24; approval date 6 November 2024). The study was prospectively registered at ClinicalTrials.gov (NCT07146295) and in the Dutch Trial Register (Landelijk Trial Register; LTR; NL-005321). The boards of directors of all participating hospitals approved local participation in the study.

Written informed consent will be obtained from all participating patients. Consent is required because the study involves prospective data collection for research purposes and because the tracer used for SLNB may differ from standard practice during the phased implementation. Patients will be informed about the study objectives, procedures, potential risks and benefits, data handling and their right to withdraw without consequences for their care.

The study is considered low risk. ICG has an established safety profile, is non-ionising and is widely used in clinical practice for other indications.^63^ Adverse events potentially related to ICG use will be monitored and recorded throughout the study. The trial will be conducted in accordance with the principles of the Declaration of Helsinki and Good Clinical Practice guidelines.

Study results will be disseminated through publication in open-access, peer-reviewed scientific journals and presentation at national and international conferences focusing on oncological surgery, breast cancer care and/or image-guided surgery. In addition, implementation products developed within the study, including a clinical protocol, implementation guide, educational materials and patient information resources, will be made publicly available to support national scale-up.

## Discussion

### Clinical impact

Fluorescence-guided SLNB using ICG offers a promising alternative to radioisotope-based techniques that may reduce patient burden, logistical complexity and healthcare costs. Despite growing evidence supporting its clinical performance and advantages over traditional tracers, uptake of ICG-guided SLNB in routine practice remains limited. This appears to reflect implementation-related challenges rather than uncertainty regarding clinical effectiveness. The INFINITE trial is designed to address this implementation gap by prospectively evaluating how ICG-guided SLNB is introduced, adopted and embedded in routine care, alongside assessment of real-world effectiveness, patient-reported experience and costs.

By embedding implementation and effectiveness evaluation within a structured, phased implementation strategy, the study aims to generate clinically relevant data that reflect variation in institutional context, surgical teams and learning curves. The inclusion of per-case and per-node effectiveness outcomes, temporal performance measures and pathological findings allows assessment of SLNB performance under real-world conditions. Implementation outcomes such as penetration and adoption describe the extent to which ICG-guided SLNB is taken up in routine practice, while measures of fidelity, appropriateness, feasibility and acceptability provide insight into why implementation succeeds or lags across sites. This integrated approach is intended to support clinicians, hospital administrators and guideline developers in making informed decisions regarding broader adoption and sustainable integration of ICG-guided SLNB.

### Strengths and limitations

The major strength of the INFINITE trial lies in its design. The hybrid effectiveness-implementation approach enables concurrent evaluation of implementation and clinical outcomes during real-world adoption. The stepped-wedge cluster design supports phased rollout, maintains transitional safety and provides repeated opportunities to evaluate and refine implementation strategies over time. The fixed transition period with dual-tracer use supports surgeon training and enables within-patient comparisons, which may strengthen confidence in diagnostic reliability during early adoption. The multicentre setting further increases generalisability by capturing variation in institutional context, geographical location and surgical volumes.

An additional strength is the use of an integrated, theory-informed implementation approach that combines a process model (Grol and Wensing), a determinant framework (CFIR) and an evaluation framework (Proctor *et al*).^48^,^50^ This allows the trial not only to measure implementation outcomes but also to systematically identify barriers and facilitators, inform the adaptation of implementation strategies and interpret observed variation in uptake and performance across sites. Embedding this approach within iterative evaluation cycles is designed to support learning during implementation while generating transferable insights for future scale-up.

Several limitations should be acknowledged. Cluster transitions are based on site readiness rather than random allocation. Although reflecting real-world implementation, a non-randomised comparison between pre- and post-implementation phases may be influenced by temporal trends or learning effects within the study period. Yet, the relatively short trial duration and stable clinical context limit the likelihood that such trends materially affect this comparison. The questionnaire-based implementation outcomes (appropriateness, feasibility and acceptability) are formally assessed at a single time point in Phase III. This timing was chosen so that providers can accumulate sufficient experience to give an informed appraisal, but it may limit characterisation of how these outcomes evolve during implementation. Some implementation outcomes, such as fidelity, rely on surgeon self-reporting and may be subject to reporting bias. The sample size is determined by feasibility rather than formal power calculations, which may limit precision for selected secondary outcomes. Although the study includes in-hospital data and collects patient-incurred costs, it is not designed to provide a full societal cost-effectiveness analysis.

Finally, the trial is also conducted in an evolving axillary management landscape, in which recent and ongoing trials suggest that axillary de-escalation, including omission of SLNB, may be safe in selected patients.^64^,^67^ However, this evidence base remains limited, and uptake in routine practice is cautious. SLNB remains indicated for many patients, underscoring the continued need for safe, efficient and consistently implemented techniques.

## Supplementary material

10.1136/bmjopen-2026-117847 online supplemental file 1
