# Supplementary material for: Fluorescence imaging using indocyanine green to identify sentinel lymph nodes during surgery for breast cancer (INFINITE): protocol for a hybrid effectiveness-implementation trial using a stepped-wedge cluster design
Source: BMJ Open. 2026 Jun 24;16(6):e117847. doi: 10.1136/bmjopen-2026-117847 (PMC13295786; doi:10.1136/bmjopen-2026-117847)
Supplement: online supplemental file 1 [file bmjopen-16-6-s001.docx]

**Online supplementary material 1**

**Fluorescent Imaging using Indocyanine Green to Identify Sentinel Lymph Nodes during Surgery for Breast Cancer (INFINITE)**

| **Contributors to the INFINITE study group** | | | | | |
| --- | --- | --- | --- | --- | --- |
| **Institution** | **Location** | **First name** | **Last name** | **Title/Academic degree** | **(Medical) Specialty** |
| St. Antonius Ziekenhuis | Utrecht | A. | Doeksen | MD, PhD | Oncological Surgery |
| St. Antonius Ziekenhuis | Utrecht | E.L. | Postma | MD, PhD | Oncological Surgery |
| St. Antonius Ziekenhuis | Utrecht | I.J. | Henskens | MD | Oncological Surgery |
| St. Antonius Ziekenhuis | Utrecht | C.F.P. | van Swol | MSc, PhD | Engineering; Clinical physics |
| St. Antonius Ziekenhuis | Nieuwegein | J. | Lavalaye | MD, PhD | Nuclear Medicine |
| St. Antonius Ziekenhuis | Nieuwegein | L.M. | Dijksman | MSc, PhD | Epidemiology; Value-Based Healthcare |
| Amsterdam University Medical Centre | Amsterdam | H.J. | Schuijt | MD, BSc; PhD | Geriatrics; Implementation Science |
| University Medical Centre Utrecht | Utrecht | H.M. | Verkooijen | Professor, MSc, PhD | Epidemiology; Evaluation of Image-Guided Treatment |
| Diakonessenhuis | Utrecht | J.H. | Volders | MD, PhD | Oncological Surgery |
| Ziekenhuisgroep Twente | Almelo, Hengelo | D.J. | Evers | MD, PhD | Oncological Surgery |
| Dijklander Ziekenhuis | Hoorn, Purmerend | L.M. | de Widt-Levert | MD | Oncological Surgery |
| Noordwest Ziekenhuis | Alkmaar,  Den Helder | G.A. | Gooiker | MD, PhD | Oncological Surgery |
| Alrijne Ziekenhuis | Leiden, Leidendorp | C.C. | van der Pol | MD, PhD | Oncological Surgery |
| Canisius Wilhelmina Ziekenhuis | Nijmegen | D.J.P. | van Uden | MD, PhD | Oncological Surgery |
| Canisius Wilhelmina Ziekenhuis | Nijmegen | M. | Keemers-Gels | MD, PhD | Oncological Surgery |
| Spaarne Gasthuis | Hoofddorp, Haarlem | K.M. | Blaauwendraat-Hans | MD | Oncological Surgery |
| Spaarne Gasthuis | Hoofddorp, Haarlem | D.A.M. | Sloothaak | MD, PhD | Oncological Surgery |
| MD, Doctor of medicine; MSc, Master of Science; PhD, Doctor of philosophy | | | | | |
