# Supplementary material for: Fluorescence imaging using indocyanine green to identify sentinel lymph nodes during surgery for breast cancer (INFINITE): protocol for a hybrid effectiveness-implementation trial using a stepped-wedge cluster design
Source: BMJ Open. 2026 Jun 24;16(6):e117847. doi: 10.1136/bmjopen-2026-117847 (PMC13295786; doi:10.1136/bmjopen-2026-117847)
Supplement: Supplementary file 2 [file bmjopen-2026-117847.reviewer_comments.pdf]

PEER REVIEW HISTORY

BMJ Open publishes all reviews undertaken for accepted manuscripts. Reviewers are asked to complete a checklist review form and are provided with free text boxes to elaborate on their assessment. These free text comments are reproduced below.

ARTICLE DETAILS

Title (Provisional)

Fluorescent Imaging using Indocyanine Green to Identify Sentinel Lymph Nodes during Surgery for Breast Cancer (INFINITE): Protocol for a Hybrid Effectiveness-Implementation Trial using a Stepped-Wedge Cluster Design

Authors

Henskens, Isabelle Juliëtte; Dinger, Tessa L.; Stekelenburg, Iza; Dijksman, Lea M.; Verkooijen, Helena M; Postma, Emily L.; Doeksen, Annemiek; Schuijt, Henk J.; Study Group, INFINITE

VERSION 1 - REVIEW

**Reviewer**	**1**
**Name**	**Lambooij, Anke**
**Affiliation**	**Instituut Verantwoord Medicijngebruik**
**Date**	**27-May-2026**
**COI**	**None**

The abstract does not contain information about the implementation models used.

p10, 33-53: The authors do not explain the basis for their selection of six implementation strategies in step 4 of the Grol and Wensing model. While these choices appear reasonable in themselves, there does not seem to have been a formal analysis of barriers and facilitators, or such an analysis is not reported.

p11, 54: Some implementation outcomes, such as acceptability, appropiateness and feasibility, may be crucial in determining surgeons' engagement during the training in phase 1 and their participation in phase 2. Did the authors consider assessing these aspects at an earlier stage in the study? These aspects could also be used to evaluate the impact of training materials used in Phase 1.

Compliments on Table 1. This table provides a clear overview of the numerous and complex outcomes that will be measured.

Ref 48: The authors refer to the first CFIR publication. Do they plan to use the original CFIR constructs or are they going to use the updated CFIR?

VERSION 1 - AUTHOR RESPONSE

Reviewer: 1

Mrs. Anke Lambooij, Instituut Verantwoord Medicijngebruik

Comments to the Author:

The abstract does not contain information about the implementation models used.

Response: We agree that naming the underpinning implementation frameworks and models strengthens the abstract. We have added a concise sentence to the 'Methods and analysis' section stating that implementation is guided by an integrated approach combining the Grol and Wensing model (process model), the Consolidated Framework for Implementation Research (CFIR; determinant framework) and Proctor's outcomes framework (evaluation framework). To accommodate this within the 300-word abstract limit, we made minor compensatory edits elsewhere without altering the content.

p10, 33-53: The authors do not explain the basis for their selection of six implementation strategies in step 4 of the Grol and Wensing model. While these choices appear reasonable in themselves, there does not seem to have been a formal analysis of barriers and facilitators, or such an analysis is not reported.

Response: We have clarified the basis for selecting the six initial implementation strategies in Step 4. The revised text now explains that strategy selection was directly informed by the Step 3 problem analysis, in which the Value Proposition Canvas was used to map the limitations of ^{99m}Tc-nanocolloid and the unmet needs of patients and providers, complemented by determinants identified in the SLNB and ICG-fluorescence literature and by input from the multidisciplinary project team. The candidate strategies were operationalised using the Expert Recommendations for Implementing Change (ERIC) project and ZonMw guidelines. We would also like to provide some additional context regarding barriers and facilitators. By design, this is undertaken predominantly as a project-based activity, reflecting the pragmatic and iterative nature of the implementation. During the three-monthly evaluation cycles (Step 7), barriers and facilitators are mapped against the CFIR domains and used for ongoing, project-level optimisation and refinement of the implementation strategies via the CFIR-ERIC matching tool. One CFIR-based assessment is embedded as a formal research component. This is embedded within the trial's mixed-methods design and comprises brief open-text questions in the healthcare provider questionnaire administered in the third month of Phase III. These responses will contextualise the quantitative outcomes. We have clarified this distinction in the 'An integrated implementation approach' section.

p11, 54: Some implementation outcomes, such as acceptability, appropriateness and feasibility, may be crucial in determining surgeons' engagement during the training in phase 1 and their participation in phase 2. Did the

authors consider assessing these aspects at an earlier stage in the study? These aspects could also be used to evaluate the impact of training materials used in Phase 1.

Response: The formal assessment of acceptability, appropriateness and feasibility is performed in the third month of Phase III. This allows providers to have gained sufficient experience to give an informed appraisal. We agree, however, that earlier indicators are valuable. As a project-based activity, participants provide anonymous feedback on the Phase I training. This feedback is used to refine the e-learning module, in-person workshop and educational materials. Early indications of acceptability and feasibility, together with provider engagement, are additionally captured through the three-monthly project-based evaluation cycles (Step 7), enabling timely identification of sites that require additional support. We have clarified this in the 'Training' and 'An integrated implementation approach' sections, and added it to the 'Discussion'. We have also added the single time-point assessment of these questionnaire-based outcomes as a fifth point in the 'Strengths and limitations of this study' section, acknowledging the trade-off between earlier measurement and informed appraisal.

Compliments on Table 1. This table provides a clear overview of the numerous and complex outcomes that will be measured.

Response: We are pleased that Table 1 provides a clear overview of the implementation, intervention and patient-reported experience outcomes and their definitions, measures and data sources.

Ref 48: The authors refer to the first CFIR publication. Do they plan to use the original CFIR constructs or are they going to use the updated CFIR?

Response: We will use the updated CFIR (Damschroder et al., 2022) rather than the original 2009 framework. We have revised the manuscript accordingly: the 'An integrated implementation approach' section and the Step 7 evaluation now refer to the updated CFIR and its five domains (innovation, outer setting, inner setting, individuals and the implementation process).

Reviewer: 1

If you have selected 'Yes' above, please provide details of any competing interests.: Not applicable
